# THOC5 controls 3′end-processing of immediate early genes via interaction with polyadenylation specific factor 100 (CPSF100)

**DOI:** 10.1093/nar/gku911

**Published:** 2014-10-01

**Authors:** Doan Duy Hai Tran, Shashank Saran, Andrew J.K. Williamson, Andrew Pierce, Oliver Dittrich-Breiholz, Lutz Wiehlmann, Alexandra Koch, Anthony D. Whetton, Teruko Tamura

**Affiliations:** 1 Institut fuer Biochemie, OE4310, Medizinische Hochschule Hannover, Carl-Neuberg-Strasse 1, D-30623 Hannover, Germany; 2 Stem Cell and Leukaemia Proteomics Laboratory, The University of Manchester, Manchester Academic Health Science Centre, 27 Palatine Road, Withington Manchester, M20 3LJ UK; 3 Pädiatrische Pneumologie OE6710 Medizinische Hochschule Hannover, Carl-Neuberg-Strasse 1, D-30623 Hannover, Germany

## Abstract

Transcription of immediate early genes (IEGs) in response to extrinsic and intrinsic signals is tightly regulated at multiple stages. It is known that untranslated regions of the RNA can play a role in these processes. Here we show that THOC5, a member of the TREX (transcription/export) complex, plays a role in expression of only a subset of constitutively active genes, however transcriptome analysis reveals that more than 90% of IEG were not induced by serum in THOC5 depleted cells. Furthermore, THOC5 depletion does not influence the expression of the most rapidly induced IEGs, e.g. *Fos* and *Jun*. One group of THOC5 target genes, including *Id1*, *Id3* and *Wnt11* transcripts, were not released from chromatin in THOC5 depleted cells. Genes in another group, including *Myc* and *Smad7* transcripts, were released with shortening of 3′UTR by alternative cleavage, and were spliced but export was impaired in THOC5 depleted cells. By interactome analysis using THOC5 as bait, we show that upon stimulation with serum THOC5 forms a complex with polyadenylation-specific factor 100 (CPSF100). THOC5 is required for recruitment of CPSF100 to 3′UTR of THOC5 target genes. These data suggest the presence of a novel mechanism for the control of IEG response by THOC5 via 3′end-processing.

## INTRODUCTION

Upon stimulation with growth factors or other stimuli such as cytokines, which are often found in serum, genes are activated that trigger cell proliferation, differentiation, apoptosis and/or cell movement. This pool of genes called immediate early genes (IEGs), are rapidly and transiently induced. Expression of IEGs is tightly regulated via the elements in promoters, RNA processing, RNA export and/or RNA stability. It is known that untranslated regions of the RNA can play a role in these processes. The 3′untranslated region (3′UTR) of mRNA can affect translation efficiency, localization and stability of the mRNA, however details of the molecular events underlying these phenomena remain unclear. The THO complex,(Suppressors of the transcriptional defects of hrp1 delta by overexpression) complex, which is a sub-member of TREX (transcription/export), was originally identified in *Saccharomyces cerevisiae* as a five protein complex (Tho2p, Hpr1p, Mft1p, Thp2p and Tex1) (1–7) that plays a role in transcriptional elongation, nuclear RNA export and genome stability. In higher eukaryotes such as *Drosophila melanogaster* (8) or humans (9), three proteins, (THOC1/hHpr1/p84, THOC2/hRlr1 and THOC3) and three additional unique proteins were identified, namely THOC5/Fms interacting protein (FMIP) (10), THOC6 and THOC7, as members of the THO complex. We have previously identified THOC5-dependent mRNAs in the fibroblast system (11). Surprisingly, only 143 genes were downregulated by depletion of THOC5. Furthermore, we recently examined THOC5-dependent mRNAs in monocytes/macrophages. In this system, only 99 genes were downregulated upon depletion of THOC5 (12); depletion of THOC5 does not affect bulk poly (A)+ RNA export (13) and it has been recently shown that knockdown of THOC5 in HeLa cells leads to downregulation of 275 genes (14). Interestingly, we have recently shown that a subset of growth factor inducible IEGs, such as *v-ets* E26 (*Ets*) family genes, but not early growth response 1 (*Egr*1), were THOC5-dependent (12), suggesting that THOC5 selected its target mRNAs via unknown element(s). In present study, we examined the role of THOC5 during IEG response. Surprisingly, induction of more than 90% of serum inducible genes was affected upon depletion of THOC5, however the most rapidly and transiently induced IEGs, such as *c-Jun, c-Fos, Ier2* or *Egr1* were independent of the modulation of THOC5 levels. We have demonstrated that THOC5 is required for the recruitment of cleavage and polyadenylation specific factor 100 (CPSF100) to the THOC5 target genes. These data indicate that IEG response is controlled by THOC5 with 3′ end-processing machinery.

## MATERIALS AND METHODS

### Cell culture and transfection

Mouse embryonic fibroblast (MEF) cells derived from Rosa26ER^T2^ Cre: Ola126 THOC5 (flox/flox) (15) and mouse NIH3T3 cells were grown in Dulbecco's modified Eagle's medium supplemented with 10% (v/v) fetal calf serum (FCS). Cells were treated with Tamoxifen (10 μM) for 24 h and then further incubated in growth medium for 24 h. After serum starvation for further 24 h, cells were stimulated with 20% serum for various times as indicated. siRNA against CPSF100 (SC-142546) and control siRNA (SC-37007) were from Santa Cruz Biotechnology Inc. (Santa Cruz, CA, USA). Transfection was performed using Lipofectamine 2000 reagent (Invitrogene, Carlsbad, CA, USA).

### Isolation of cytoplasmic, nucleoplasmic and chromatin associated RNA fractions

Isolation of RNA fractions was performed as described previously (11).

Cells (1 × 10^7^) were lysed by cytoplasmic lysis buffer (50-mM TrisHCl, pH 8.0, 140-mM NaCl, 1.5-mM MgCl_2_, 0.5% NP40, RNase inhibitor, 1-mM Dithiothreitol (DTT)). After centrifugation (800 × *g* for 2 min) supernatant (cytoplasmic fraction) was removed and the pellet was resuspended in 40-μl nucleus resuspension buffer (20-mM TrisHCl, pH 7.9, 75-mM NaCl, 0.5-mM ethylenediaminetetraacetic acid (EDTA), 0.125-mM PMSF, 0.1-μg/μl tRNA, 50% Glycerol). Two-hundred microliter of NUN (NaCl/Urea/NP40) buffer (20-mM HEPES, pH 7.6, 1-mM DTT, 7.5-mM MgCl_2_, 0.2-mM EDTA, 0.1-μl/μl tRNA, 0.3-M NaCl, 1-M Urea, 1% NP40) was then added to the suspensions. After centrifugation, supernatant was collected as nucleoplasmic fraction. The pellet was incubated with DNase I for 10 min at room temperature (chromatin associated fraction). RNA was isolated from each fraction using High Pure RNA Isolation Kit (Roche Diagnostics) according to the manufacture's instructions.

### Semi-quantitative RT-PCR and qRT-PCR analysis

RNA was isolated from MEF cells with the High Pure RNA Isolation kit (Roche Diagnostics, Mannheim, Germany) according to the manufacturer's instructions. One microgram of RNA was reverse-transcribed using oligo dT primers and the Omniscript reverse transcriptase kit (Qiagen, Hilden, Germany) following the instructions provided. One-twentieth of the cDNA mix was used for real-time polymerase chain reaction (PCR) using 10 pmol of forward and reverse primer and SensiFAST SYBR No-ROX kit (Bioline, London, UK) in a Qiagen Rotorgene machine. Cytoplasmic RNA was isolated from cells as previously described (11). Primer pairs for each PCR are described in Table 1.

**Table 1.** PCR primer pair sequences for selected genes

Gene	Forward primer	Reverse primer	RT-PCR	qRT-PCR	ChIP
*C8orf4*	CTATGTTTCTACAGGATTTGTAC	CCATATTCATTTCCTAATGCTTAC	x		
*c-fos*	TTCCTGGCAATAGCGTGTTC	TTCAGACCACCTCGACAATG	x	x	
*c-jun*	TGTGCCCCAAGAACGTGAC	CCGGGTTGAAGTTGCTGAG	x	x	
*Dusp2*	TGTGGAAATCTTGCCCTACCT	CCCACTATTCTTCACCGAGTCTA	x		
*Egr1*	AGCGAACAACCCTATGAGCAC	TCGTTTGGCTGGGATAACTCG	x	x	
*Egr1 (uncleaved)*	GTACTTGTGTTTGCTTAAACAAAGTAAC	ACCACACATGGGTAAGGACTCA	x		
*Errfi1*	GCTGCTCAGGATATTCGAGTC	CCAACAGTTGTTAGGTGCTCC	x		
*Gapdh*	AGGTCGGTGTGAACGGATTTG	TGTAGACCATGTAGTTGAGGTCA	x		
*Gapdh (uncleaved)*	AGCATCTCCCTCACAATTTCCATC	GTTGATTGAGCCTGCTTCACCTC	x	x	
*Hes1*	CCAGCCAGTGTCAACACGA	AATGCCGGGAGCTATCTTTCT	x		
*Id1*	CTGAACTCGGAGTCTGAAGT	ACTTTTTTCCTCTTGCCTCCT	x	x	
*Id1 (uncleaved)*	GGTCACATTTCGTGCTTCTCG	AAACACTCATTCAGGTCGGTAAG	x		
*Id3*	CGCATCTCCCGATCCAGACA	CTGGGTTAAGATCGAAGCTCATCC	x	x	
*Id3 (uncleaved)*	CAGGAAGGTGACTTTCTGTAATC	CAGCCCTTCCTACTAACCAAG	x	x	
*Id3 3‘UTR*	CTGATTATGAACTCTATAATAG	CAAAGTGTTCAAAAATGGTTTATT		x	x
*Id3 Promoter*	CCTCCAGAAAAGGCATATTC	GAATGAGGAAGCGCTGATAC		x	x
*Ier2*	TTTGAGCGACGGTAGTGATGC	GAGACTGGAGAAGCGCCTTTG	x	x	
*Ier2 (3*′*UTR)*	CTGGTCGTAGTTGCTGCCGTAG	GCGTTTTACACCGATGGTCTTTATTTTCC		x	x
*Ier2 (Promoter)*	TGCGGAAAACTGGGAGATCTTTAAC	GATTATACTATGGCGAAAGGATCAAC		x	x
*Ier2 (uncleaved)*	CTGGTCGTAGTTGCTGCCGTAG	CAGTCAAACCAGTTCCGGAG	x		
*Lif*	GCTGTATCGGATGGTCGCATA	CACAGACGGCAAAGCACATT	x		
*Myc*	GCTGGATTTCCTTTGGGCGT	CGCAACATAGGATGGAGAGCA	x		
*Myc (2071–2245)*	GGCTTTGGGACTGTAAGCTTCAGC	GGCCCTATTTACATGGGAAAATTGGATAG	x		
*Myc (2071–2399)*	GGCTTTGGGACTGTAAGCTTCAGC	GTATTTTTTCCAATTATTTTATTTTTTTCTAAAAAC	x		
*Smad7*	GCATTCCTCGGAAGTCAAGAG	CCAGGGGCCAGATAATTCGT	x		
*Smad7 (3977–4128)*	GCTCGCTCGTATGATACTTTGAC	CCTTTCCTCTCTCAAAGCACTAC	x		
*Smad7 (3977–4389)*	GCTCGCTCGTATGATACTTTGAC	CATTCAGCTAGGTGATAACACCCA	x		
*Snai1*	CAAGGAGTACCTCAGCCTGG	GGTCAGCAAAAGCACGGTT	x		
*Wnt11*	GGTGGTACACCGGCCTATG	TCACTGCCGTTGGAAGTCTTG	x	x	
*Wnt11 (unlceaved)*	CTGTTTGTGATGTCTGCCAATAG	GAAGATCTGCCTAAGACACGAAAG	x		
*Zfp36*	CATGGATCTCTCTGCCATCTAC	GAGCCAAAGGTGCAAAACCA	x		

**C8orf4** (NM_026931.2): RIKEN cDNA 1810011O10 gene (191nt); **c-fos** (NM_010234.2): FBJ osteosarcoma oncogene (169 nt); **c-jun** (NM_010591.2): jun proto-oncogene (243 nt); **Dusp2** (NM_010090.2): dual specificity phosphatase 2 (232 nt); **Dusp2** (NM_010090.2): dual specificity phosphatase 2 (232 nt); **Egr1** (NM_007913.5): early growth response 1 (100 nt); **Egr1 (uncleaved)** (229 nt); **Errfi1** (NM_133753.1): ERBB receptor feedback inhibitor 1 (182 nt); **Gapdh** (NM_008084.2): glyceraldehyde-3 phosphate dehydrogenase (123 nt); **Gapdh (uncleaved)** (176 nt); **Hes1** (NM_008235.2): hairy and enhancer of split 1 (166 nt); **Id1** (NM_010495.3): Inhibitor of DNA binding 1 (203 nt); **Id1 (uncleaved)** (285 nt); **Id3** (NM_008321.2): Inhibitor of DNA binding 3 (202 nt); **Id3 (uncleaved)** (214 nt); **Id3 (3′UTR)** (139 nt); **Id3 (Promoter)** (116 nt); **Ier2** (NM_010499.4): immediate early response 2 (173 nt); **Ier2 (3′UTR)** (174 nt); **Ier2 (Promoter)** (167 nt); **Ier2 (uncleaved)** (261 nt); **Lif** (NM_008501.2): leukemia inhibitory factor (156 nt); **Myc** (NM_001177352.1): myelocytomatosis oncogene (274 nt); **Myc (2071–2245)** (175 nt); **Myc (2071–2399)** (329 nt); **Smad7** (NM_001042660.1): SMAD family member 7 (225 nt); **Smad7 (3977–4128)** (152 nt); **Smad7 (3977–4389)** (413 nt); **Snai1** (NM_011427.2): snail family zinc finger 1 (179 nt); **Wnt11** (NM_009519.2): wingless-related MMTV integration site 11 (183 nt); **Wnt11 (uncleaved)** (215 nt); **Zfp36** (NM_011756.4): zinc finger protein 36 (218 nt).

### Chromatin precipitation

TAP-THOC5 or CTAP (C-terminal Tandem Affinity Purification) control (Stratagene, La Jolla, CA) was expressed in mouse NIH3T3 cells, or ER^T2^THOC5 (flox/flox) MEF cells were treated with or without tamoxifen for 2 days. After 24 h serum starvation, cells were stimulated with or without serum for 1 h. Cells were cross-linked by adding formaldehyde for a final concentration of 1% for 10 min at 37°C in the presence of 5% CO_2_. The reaction was stopped by adding glycine to a final concentration of 125 mM.

Cells were resuspended in 1 ml of RIPA buffer (150-mM NaCl, 1% NP40, 0.1% Sodium dodecyl sulphate, 50-mM Tris–HCl, pH 8.0, 0.2-mM EDTA) and sheared into 500-bp DNA fragments using Covaris AFA™ (Adaptive Focused Acoustics, Woburn, MA, USA) technology according to the manufacturer's instructions. Aliquots of extracts were precipitated by streptavidin beads (TAP-THOC5) or pre-coated Protein G Agarose-PLUS (Santa Cruz Biotechnology Inc.) with anti CPSF100 (Novus biologicals Inc., Littleton, CO, USA), CFIm68 (Benthyl Laboratories. Inc., Montgomery, TX, USA) or RNA polymerase II (Santa Cruz Biotechnology Inc.) antibodies or control IgG. Following 4 h or overnight rotation at 4°C, the beads were washed three times in RIPA buffer, and two times in wash buffer (500-mM NaCl, 1% NP40, 100-mM Tris–HCl at pH 8). Cross-links were reversed for 5 h at 65°C (250 mM-NaCl). One microliter of RNase A (100 mg/ml) was added to the bead suspension and incubated for 10 min at RT. Following proteinase K digestion at 55°C for 1 h, the bound DNA fraction was isolated using NucleoSpin Extract II (Macherey-Nagel, Dueren, Germany). PCR was then performed using *Id3* or *Ier2* gene-specific primers as shown in Table 1 (ChIP).

### Mapping 3′ mRNA termini

Mapping of 3′ mRNA termini has been described by Russo *et* *al*. (16). Briefly, 1 μg of RNAs was used for the cDNA synthesis. Reverse transcription was carried out using GACTCGAGTCGACATCGA(T)18(N) primers (N: A, C or G) and the Omniscript reverse transcriptase kit (Qiagen, Hilden, Germany) following the instructions provided. Briefly, the reverse transcription mixture was incubated at 95°C for 2 min. After that, reverse transcriptase was added to the reaction and incubated for 1 h at 37°C. PCR was performed with reverse primer GACTCGAGTCGACATCGATT and gene-specific forward primer. PCR products were separated by gel electrophoresis and isolated using NucleoSpin Extract II (Macherey-Nagel). These isolated PCR products were cloned into pGEM-T vector (Promega, Madison, WI, USA) and sequenced.

### Analysis of THOC5 interacting proteins

C-terminal TAP-tagged THOC5 constructs were expressed in HEK293 cells. After serum starvation for further 24 h, cells were stimulated with 20% serum for 1 h. Cell extracts were incubated with RNaseT1 and then precipitated by streptavidin beads and bound proteins were subjected to electrophoresis on 10% (w/v) polyacrylamide gels and stained with simply Blue Coomassie safe stain. Protein bands were excised and destained with repeated incubation in 200-mM ammonium bicarbonate, 40% (v/v) acetonitrile. Gel pieces were dried with three washes in acetonitrile and then trypsinized (trypsin resuspended in 100-mM ammonium bicarbonate, 5% (v/v) acetonitrile) overnight at 37°C. Peptides were extracted from the gel pieces by incubation in 50% (v/v) acetonitrile, 0.1% (v/v) formic acid and peptides were desiccated and resuspended in 3% (v/v) acetonitrile, 0.1% (v/v) formic acid, 20-mM citric acid, pH 2.7. For each analysis, 10% of the peptide sample was loaded onto a nanoACQUITY UPLC Symmetry C18 Trap (5 μm, 180 μm × 20 mm), and flow was set to 15 μl/min of 3% (v/v) acetonitrile, 0.1% (v/v) formic acid and 20-mM citric acid for 5 min. Analytical separation of the peptides was performed using a nanoACQUITY UPLC BEH C18 column (1.7 μm, 75 μm × 250 mm). Briefly, peptides were separated over a 91-min solvent gradient from 3% (v/v) acetonitrile, 0.1% (v/v) formic acid to 40% (v/v) acetonitrile, 0.1% (v/v) formic acid online to a LTQ Orbitrap Velos (Thermo). Data were acquired using an information-dependent acquisition method where, for each cycle one full MS scan of m/z 300–1700 was acquired in the Orbitrap at a resolution of 60 000 at m/z 400 with an automatic gain control target of 106. Each full scan was followed by the selection of the 20 most intense ions; CID (collision-induced dissociation) and MS/MS analysis was performed in the LTQ Orbitrap Velos instrument. Selected ions were excluded from further analysis for 60 s. Ions with an unassigned charge or a charge of +1 were rejected.

Data were analyzed using Mascot (Matrix Sciences) software; the parameters were: Uniprot database, trypsin with up to one missed cleavage allowed, variable modifications oxidized methionine, phosphorylated serine, threonine and tyrosine modifications permitted and peptide tolerance of 0.025 and 0.03 Da for MS/MS tolerance. A functional annotation of interacting proteins was carried out using the DAVID software package (Version 6.7).

### Immunoblot procedures

Details of immunoblotting have been described previously (17). Monoclonal antibody against Lamin AC and polyclonal antibody against Histone H3 were from Cell Signaling Technology (Beverly, MA, USA). Monoclonal antibody against GAPDH and Polyclonal antibody against Actin were purchased from Santa Cruz Biotechnology Inc., and a monoclonal antibody against THOC5 was generated as described previously (18).

### Microarray-based mRNA expression analysis

Dual-color microarray experiments based on the Agilent platform have been performed as described previously (12). The complete microarray data along with processing protocols have been deposited in NCBI's Gene Expression Omnibus and are accessible through GEO series accession number GSE59561. In order to identify serum-inducible genes in control cells, whole processed data were filtered as follows: (i) Ratio of relative gene expression (1 h serum-stimulated/unstimulated) > 4-fold (rounded to one decimal place); (ii) Normalized processed signal intensity of serum-stimulated (red) channel > 200; (iii) Sufficient degree of annotation of respective transcripts as assessed by Ingenuity Pathway Analysis.

## RESULTS

### The most rapidly and transiently induced IEGs are THOC5 independent

**Figure 1.**
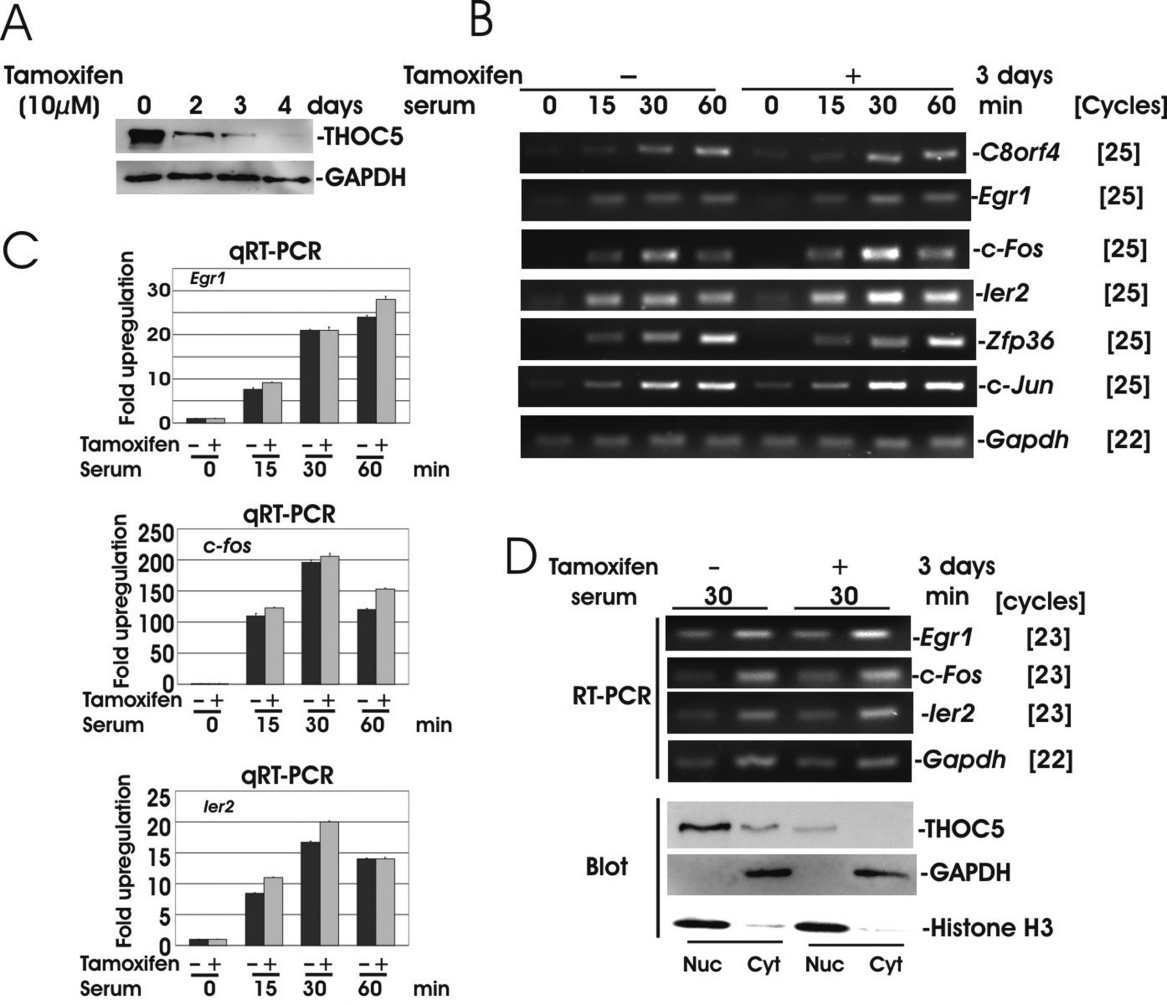
The most rapidly and transiently IEGs are not THOC5 dependent. (**A**) ER^T2^Cre THOC5 (flox/flox) MEF cells were treated with tamoxifen (10 μM) for 0–4 days as indicated. Total cell lysates were applied to THOC5- and GAPDH-specific immunoblot. (**B** and **C**) ER^T2^Cre THOC5 (flox/flox) MEF cells were treated with or without tamoxifen for 2 days and prior to incubation in medium without serum for 24 h. The cells were then stimulated with 20% serum for 0, 15, 30 and 60 min. Total RNA was isolated from each sample and semi-quantitative RT-PCR (B) and quantitative (q)RT-PCR (C) were performed. (**D**) Cells were prepared as described in (B and C) and stimulated with 20% serum for 30 min. RNA was isolated from the nuclear (Nuc) and cytoplasmic (Cyt) fractions and analyzed by RT-PCR. Fractionation quality was measured by immunoblot analysis of THOC5, GAPDH and Histone H3 (Blot). Three independent experiments were performed.

We have recently established a mouse embryonic fibroblast (MEF) cell line from tamoxifen-inducible THOC5 knockout (Rosa26ER^T2^ Cre: THOC5 (flox/flox)) mice (12,15). In this cell line, the THOC5 protein level was reduced by 90% within 3 days after incubation with tamoxifen (Figure 1A) (12). To compare the IEG response in the presence or absence of THOC5, total RNAs were isolated from unstimulated or serum stimulated THOC5 depleted cells or control cells and subjected to transcriptome analysis. In order to identify THOC5-dependent transcripts, whole data were subjected to a multistep filtering procedure as described in ‘Materials and Methods’ section. Using this filter, a total of 101 genes were upregulated more than 4-fold in the presence of THOC5 (Table 2, Supplementary Table S1), while only eight genes were upregulated to a similar extent in the absence of THOC5 (Table 2 [a]). We first examined five genes which were similarly increased in the presence or absence of THOC5 and found a similar kinetics of response to serum (Figure 1B and C). These entities were exported to cytoplasm in the absence of THOC5 (Figure 1D). Six of these genes, including *c-Jun*, were previously described as ‘the most rapidly and transiently induced IEGs’ (19,20). Modulation of *c-Jun* levels by serum was not impaired by depletion of THOC5 (Figure 1B). Notably, THOC5-independent IEGs do not contain an intron (*Ier2, C8orf4, c-Jun*) or they are co-transcriptionally spliced (*Fos, FosB, Egr1, Zfp36, Btg2*) (19).

**Table 2.** List of THOC5 dependency of serum inducible genes

Fold reduction of upregulation [group]	Gene symbol
<1.3 fold (8 genes) [a]	Aoc3, Btg2, C8orf4, Egr1, Fos,Fosb, Ier2, Zfp36
1.3–2 fold (18 genes) [b]	Arc, Atf3, Ccno, Cxcl1, Cyr61, Dnajb1, Dusp6, Egr2, Gem, Lsmem1, Nppb, Nr4a1, Rcan1, Rgs16, Sgk1, Skil, Tnfaip3, Trib1
>2 fold (75 genes) [c]	A3galt2, Agbl3, Apobec1, Apol8, Areg, Arl5b, Bhlhe40, C4orf26, Ccrn4l, Cdkn1a, Clcf1, Cmtm4, Csf2, Csf3, Csrnp1, Ctgf, Cul9, Dlx2, Dusp2, Dusp4, Dusp5, Ereg, Errfi1, F3, Fes, Foxc2, Gzmm, Hapln3, Has1, Hbegf, Hes1, Id1, Id2, Id3, Id4, Ier3, Ier5, Ifrd1, Il11, Inhba, Itga5, Junb, Kctd11, Kdm6b, Klf10, Klf9, Klhl21, Lce1f, Lce1h, Lce1i, Lif, Maff, Mmp10, Myc, Myo15a, Nfatc1, Npas4, Pdgfa, Penk, Phlda1, Proca1, Ptgs2, Ret, Serpinb2, Serpine1, Smad7, Snai1, Sox9, Sphk1, Spry4, Tmem88, Tmem95, Usp17l24, Vegfa, Wnt11

Accession numbers of each gene described in Supplementary Table S1.

### 3′end-cleavage of *Id1, Id3* or *Wnt11* genes was impaired upon depletion of THOC5

**Figure 2.**
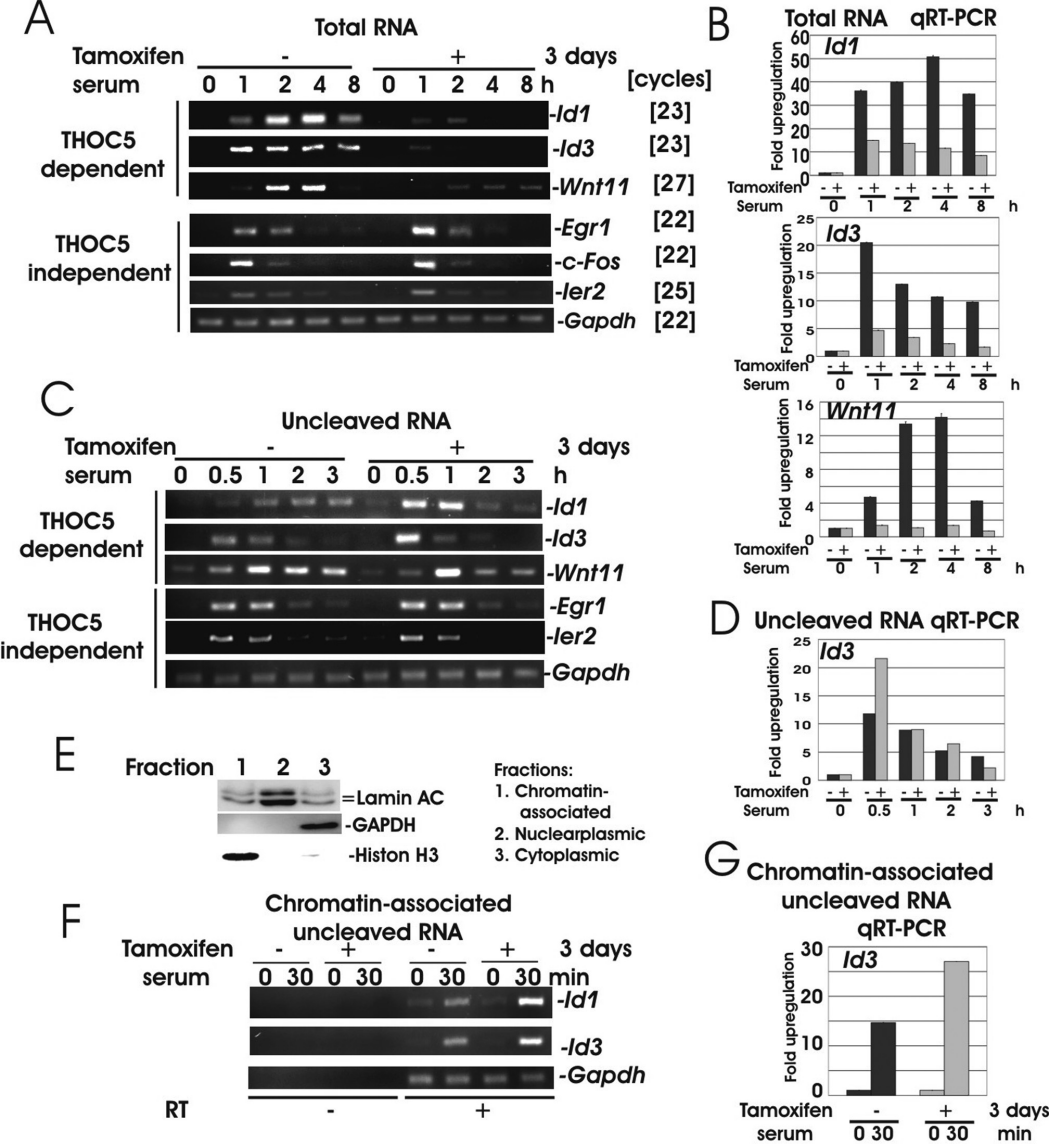
Upon depletion of THOC5, *Id1, Id3* and *Wnt11* genes were transcribed, but not released from chromatin. (**A** and **B**) ER^T2^Cre THOC5 (flox/flox) MEF cells were treated with or without tamoxifen for 2 days prior to incubation in medium without serum for 24 h. The cells were then stimulated with serum for 0, 1, 2, 4 and 8 h. Total RNA was isolated from each samples and semi-quantitative RT-PCR (A) and quantitative (q)RT-PCR (B) were performed. Primers were located in different exons (Table 1). (**C** and **D**) Cells were treated as described in (A) but stimulated with serum for 0.5, 1, 2 and 3 h. Nuclear RNA were isolated and used for RT-PCR. Primers were shown in Table 1. (**E**) Proteins were extracted from chromatin associated nucleoplasmic and cytoplasmic fractions and were used for LaminAC, GAPDH and Histone H3-specific immunoblot. (**F** and **G**) Chromatin associated RNAs were isolated from cells stimulated for 30 min with serum in the presence (Tamoxifen−) or absence (Tamoxifen+) of THOC5 and RT-PCR was performed using the same primers as (C and D). Three independent experiments were performed.

**Figure 3.**
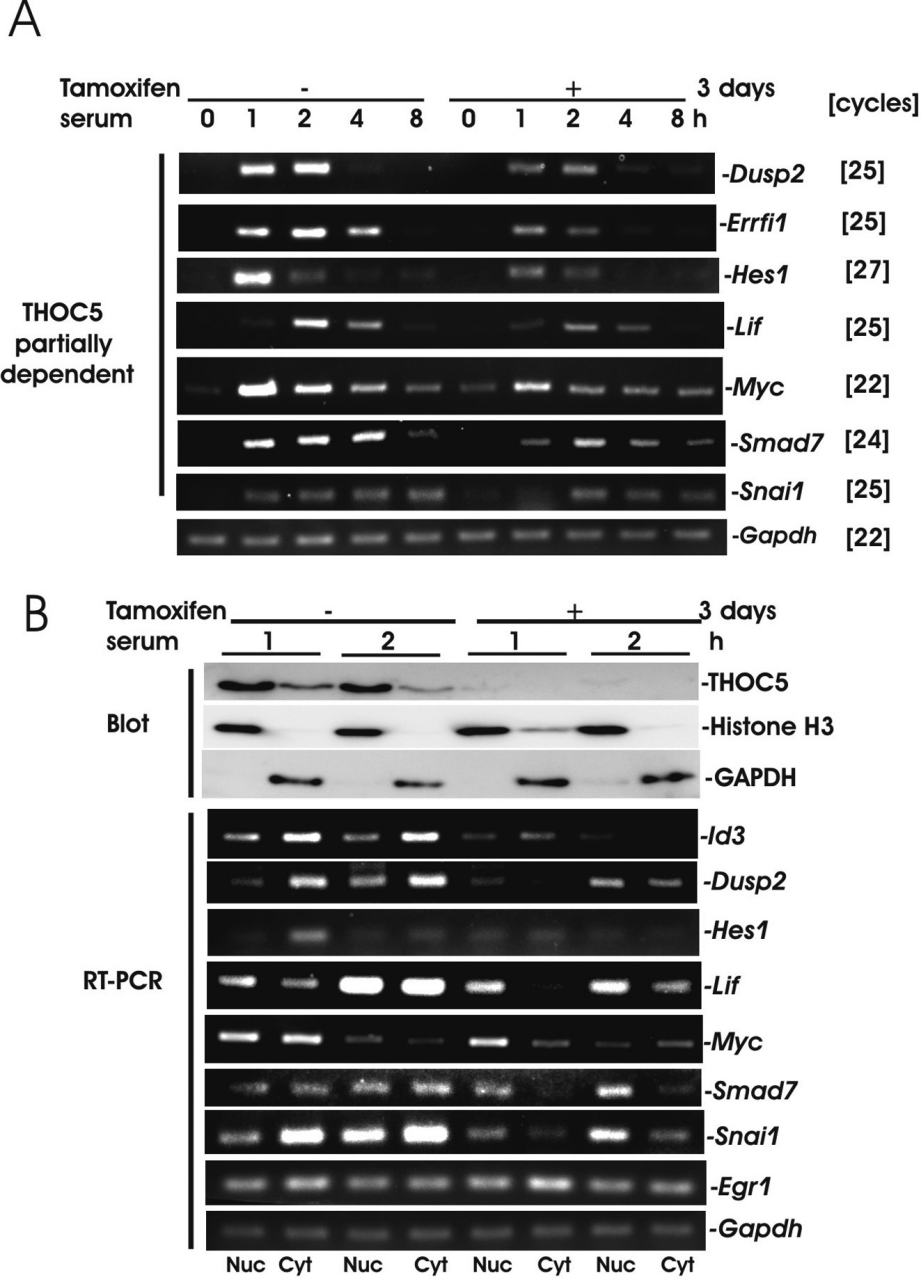
Most IEGs were THOC5 dependent, however the redundant backup pathways partially rescue their upregulation. (**A**) ER^T2^Cre THOC5 (flox/flox) MEF cells were treated as described in Figure 2A and semi-quantitative RT-PCR were performed as indicated. Primers were located at different exons (Table 1). (**B**) Protein and total RNAs were isolated from nuclear and cytoplasmic fractions after 1 or 2 h serum stimulation. Proteins were used for THOC5, Histone H3 or GAPDH-specific immunoblot (Blot) and RNAs were used for RT-PCR as indicated. Three independent experiments were performed.

We next investigated the IEGs, whose upregulation was reduced in the absence of THOC5. We selected 25 genes from those that displayed a >50% reduction in gene induction upon depletion of THOC5 (Table 2, Group [c]). To eliminate genes which are increased in expression indirectly, ER^T2^Cre THOC5 (flox/flox) MEF cells were treated with and without cycloheximide then stimulated with serum. Sixteen genes were still enhanced in expression in the presence of cycloheximide (Supplementary Figure S1). We further analyzed kinetics of upregulation of 10 genes, namely *Id1, Id3, Wnt11* (Figure 2), *Dusp2, Errfi1, Hes1, Lif, Myc, Smad7* and *Snai1* (Figure 3) in the presence or absence of THOC5. Quantitative RT-PCR was performed using primers that locate at different exons (Table 1). Among these genes, *Id1, Id3* and *Wnt11* mRNAs were not increased in expression at any time points in THOC5-depleted cells (Figure 2A and B), indicating that THOC5 is indispensable for the processing of these mRNAs. Katahira *et* *al*. (14) recently showed that THOC5 plays a role in 3′end-processing of THOC5 target genes in HeLa cells. We next examined increased expression of uncleaved forms of *Id1*, *Id3* and *Wnt11* mRNAs upon serum stimulation. Primer pairs were designed that locate up- and down-stream of cleavage sites based on the NCBI data base (Table 1). As shown in Figure 2C, upon depletion of THOC5, the uncleaved *Id1*, *Id3* and *Wnt11* mRNAs were upregulated to an even greater level than they were in the presence of THOC5. To confirm these data, we next isolated chromatin associated mRNAs (Figure 2E) and semi-quantitative RT-PCR was performed. In the absence of THOC5, chromatin associated *Id1* and *Id3* mRNAs accumulated within 30 min (Figure 2F). Uncleaved and chromatin associated *Id3* mRNA was increased more than 2-fold in the absence of THOC5 within 30 min (Figure 2D and G, qRT-PCR), suggesting that these genes were transcribed, but that the mRNA was not cleaved in the absence of THOC5.

### Redundant backup pathways only partially rescued the transcription and splicing of most IEG in the absence of THOC5

Upon deletion of THOC5, most IEGs, such as *Dusp2, Errfi1, Hes1, Lif, Myc, Smad7* and *Snai1* were upregulated at a reduced level compared to control cells but with similar kinetics to those in control cells (Figure 3A). We next examined the export of these mRNAs. The nuclear and cytoplasmic RNA were isolated from THOC5 depleted cells or control MEF cells before or after serum stimulation. To validate the quality of the nuclear and cytoplasmic fractions, the marker proteins GAPDH (cytoplasmic fraction) and Histone H3 (nuclear fraction) were employed plus THOC5*-*specific assay immunoblotting (Figure 3B). Both fractions were standardized in regard to *Gapdh* mRNA expression level, and then spliced *Dusp2, Hes1, Lif, Myc, Smad7* and *Snai1* mRNAs were examined by semi-quantitative RT-PCR using primers that locate at different exons (Table 1). As shown in Figure 3B, these RNAs were not increased in expression to the same level, and they accumulated in the nucleus. Thus, the export of *Dusp2, Hes1, Lif, Smad7* and *Snai1* mRNAs was clearly reduced (Figure 3B). These data suggest that a redundant backup pathway partially rescued the upregulation of these genes. We next examined the 3′end-cleavage sites of these genes in the presence or absence of THOC5.

### 3′end-cleavage sites of *Myc*, and *Smad7* transcripts were different in the presence or absence of THOC5

**Figure 4.**
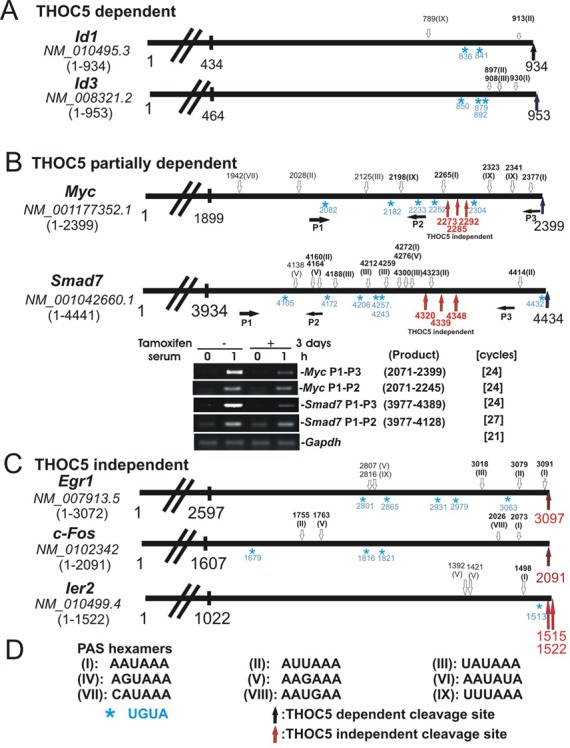
In the absence of THOC5, processing of *Myc* or *Smad7* genes was partially rescued by a backup pathway but cleaved ∼100 nt upstream from the cleavage site in the presence of THOC5. Schematic presentation of 3′UTR of several IEGs and their accession numbers (italic). Numbers represent nucleotide numbers without poly A. . PAS hexamers (white arrows; numbers represent the first nucleotide of PAS sequence shown in (**D**). Common PAS hexamers in mouse and human were shown in bold and the UGUA element was shown ‘blue *’. THOC5 dependent cleavage sites are presented by black arrows; THOC5 independent cleavage sites are represented by red arrows. (**A**) 3′UTR of THOC5 dependent genes, *Id1* and *Id3.* (**B**) 3′UTR of *Myc* and *Smad 7* genes: both genes contain THOC5 independent cleavage sites. Semi-quantitative *Myc* and *Smad7-*specific RT-PCR using up- (P1–P2) or down- stream (P1–P3) of THOC5 independent cleavage sites in the presence or absence of THOC5. (**C**) THOC5 independent gene (the most rapidly induced IEG). (D) Sequences of PAS hexamers that were indicated (21) in (A–C) as I-IX.

We next isolated *Id1, Id3, Myc, Smad7, Egr1*, *c-Fos* and *Ier2* mRNAs and analyzed the cleavage and polyadenylation sites in the presence or absence of THOC5 as previously described (16). In the presence of THOC5 all mRNAs except *Smad7* and *Egr1* are identical to the sequences published in NCBI (RefSeq numbers are shown in Figure 4). We detected *Smad7* with the seven nucleotides (nt) shorter form (Figure 4B) and *Egr1* with the 25 nt longer form (Figure 4C) exclusively. These may be due to differences in cell type. Upon knockdown of THOC5, no transcript of *Id1* or *Id3* was detected, however we detected both *Myc* and *Smad7* mRNA*s* with ∼100 nt shorter forms (arrows in red) than those in the presence of THOC5 (arrows in black) (Figure 4B). We confirmed these data by semi-quantitative RT-PCR using primers that are located up- or down-stream of THOC5-independent cleavage sites (Figure 4B). Knockdown of THOC5 did not influence the cleavage site of *Egr1*, *c-Fos* and *Ier2* (Figure 4C, arrows in red). Recently, Katahira *et* *al*. (14) proposed that THOC5 affects alternative polyadenylation site choice by recruiting cleavage factor I of constitutively active genes in HeLa cells. To examine whether the alternative polyadenylation site is one of the elements for THOC5 dependency of IEGs, we next analyzed the positions of potential polyadenylation signal (PAS) hexamers (Figure 4, arrows in white) (21) and the ‘UGUA’ element (Figure 4, blue *) in the last 500 nt of 3′UTR of these genes. We did not find any substantial differences between THOC5-dependent and -independent IEGs regarding the type of PAS hexamers (Figure 4D). For example, *Id3, Myc, Ier2* and *c-Fos* contain PAS ‘AAUAAA’ (I), while *Id1, Smad7* and *Egr1* contain PAS ‘AUUAAA’ (II). Notably, *Myc* and *Smad7* (THOC5 partially dependent) genes contain 8 and 11 PAS, respectively, while *Id1* and *Id3* (THOC5 tightly dependent) genes contain only 2–3 PAS in the last 500 nt (Figure 4A, and B).

### THOC5 interacts with CPSF100

The 3′end-processing machinery is composed of ∼20 core factors, representing CPSF, cleavage stimulation factor (CstF), cleavage factors I and II (CFIm, CFII) and poly(A) polymerase. In addition, many associated factors are responsible for the cleavage and polyadenylation reaction (22,23).

**Figure 5.**
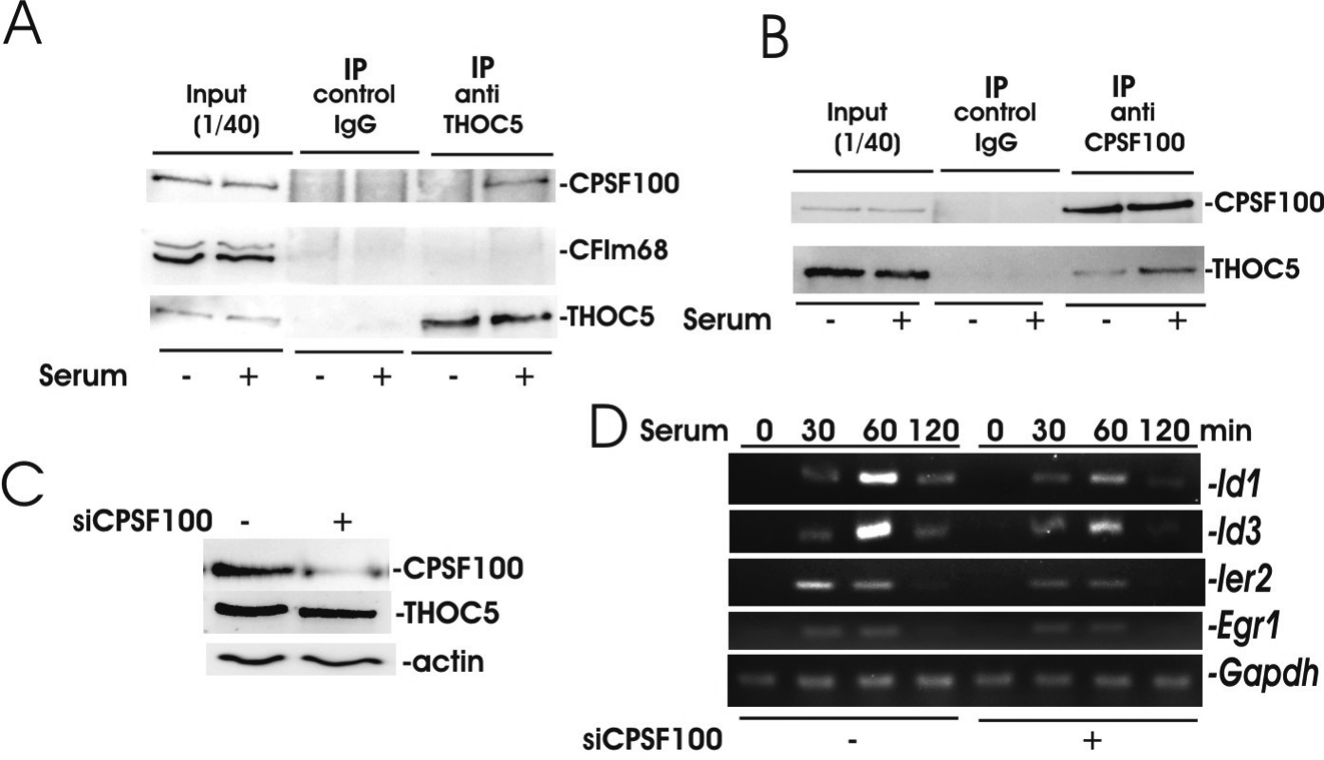
Upon stimulation with serum CPSF100 forms a complex with THOC5. (**A** and **B**) ER^T2^Cre THOC5 (flox/flox) MEF cells were serum starved for 24 h and then stimulated with or without serum for 1 h. Nuclear fractions were isolated using RNase and then precipitated with antibody against THOC5 (A), or CPSF100 (B). Immunoprecipitates were analyzed by THOC5, CPSF100 or CFIm68-specific immunoblot. (**C**) Mouse NIH3T3 cells were transfected with CPSF100-specific siRNA and control siRNA and cell lysates were used for THOC5 and CSPF100-specific immunoblot. Actin was used as a loading control. (**D**) Sister cultures from (C) were incubated without serum for 24 h, and then cells were stimulated with serum for 30, 60 or 120 min. RNAs were isolated from each sample and semi-quantitative RT-PCR was performed using *Id1, Id3, Ier2*, *Egr1* and *Gapdh*-specific primers (Table 1). Three independent experiments were performed and an example of representative data is shown here.

To obtain further insight into the molecular function of THOC5 in 3′end-processing of IEGs, we performed an interactome analysis on THOC5 with the specific remit of searching for protein members of the 3′end-processing machinery that interact with THOC5 upon serum-stimulation. In supplementary Supplementary Table S3, we provide details of proteins that were observed using mass spectrometry in a TAP (tandem affinity purification, Stratagene, La Jolla, CA, USA)–THOC5 complex from serum treated cells specifically. It was of major interest that this interactome analysis revealed that upon stimulation with serum THOC5 formed a complex with CPSF100 in the presence of RNaseT1. We confirmed the interaction between THOC5 and CPSF100 by co-immunoprecipitation using THOC5 or CPSF100 antibodies (Figure 5A and B). CFIm68 was not precipitated with THOC5 before and after serum stimulation (Figure 5A), suggesting that the role of THOC5 in regulation of 3′end-processing of IEG may be different than in constitutively active genes in HeLa cells (14).

We next examined the role of CPSF100 in 3′end-processing of IEGs. The amount of CPSF100 was reduced to ∼50% by transfection with siRNA against CPSF100 (Figure 5C). In these cells serum enhanced expression of both THOC5-dependent or independent IEGs were reduced (Figure 5D), indicating that this factor plays an essential role in 3′end-processing of THOC5-dependent and -independent genes. We next examined whether THOC5 regulates the recruitment of CPSF100 to those mRNA species that can be defined as THOC5 target IEGs from THOC5 depletion studies.

### THOC5 is required for recruiting of the CPSF100 to 3′end of THOC5 target gene

**Figure 6.**
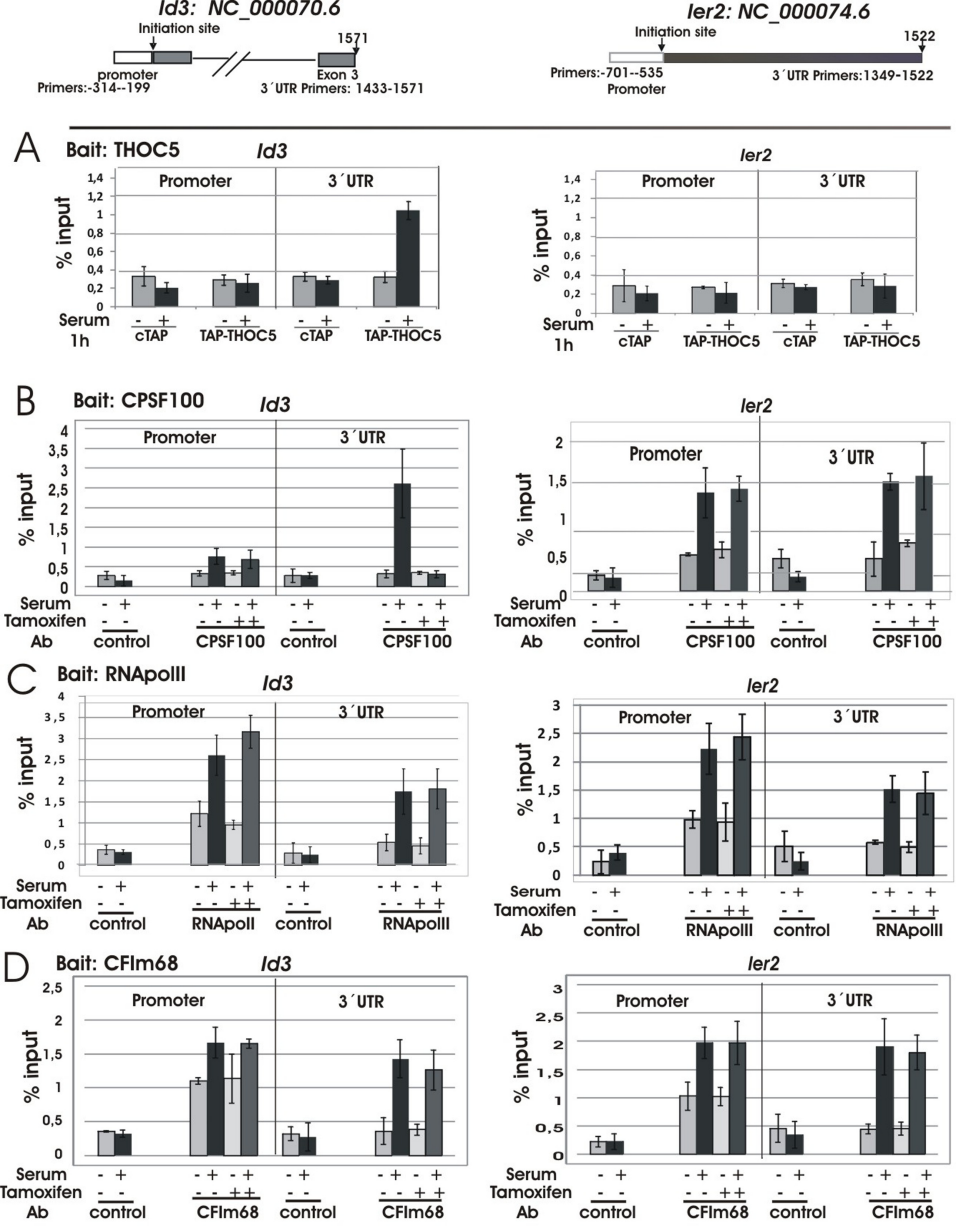
THOC5 is required for recruiting of the CPSF100 to 3′end of THOC5 target gene. pCTAP (cTAP) and pCTAP carrying THOC5 cDNA (TAP-THOC5) were transfected into mouse NIH3T3 cells (Bait: THOC5 (**A**)), or ER^T2^ THOC5 (flox/flox) MEF cells were treated with or without tamoxifen for 2 days (Baits: CPSF100 (**B**), RNApolymerase II (**C**), or CFIm68 (**D**)). The cells were then incubated for 24 h in the presence of 20% FCS. After serum starvation for 24 h, cells were stimulated with (+) or without (−) serum for 1 h. After cross-linking by adding formaldehyde, protein and DNA were extracted and the chromatin was sheared by sonication. Cell extracts and binding fractions with streptavidin Sepharose or immunoprecipitates using CPSF100, RNApolymerase II or CFIm68 antibodies or control IgG were analyzed by *Id3* (promoter region (-314- -199)and 3′UTR in exon 3 (1433-1571)) and *Ier2* (promoter region (-701- -535) and 3′UTR region (1349-1522))-specific PCR (Table 1; ChIP). The promoter region of each gene was described by Zhao *etal*. (24). Numbers represent nucleotide numbers from the initiation site for each gene. Data represent% input of each PCR reaction. Three independent experiments were performed.

We have previously shown that THOC5 is recruited to the last exon, but not the promoter region of the *Ets* gene induced by stimulation with macrophage-colony stimulating factor (M-CSF) (12). In agreement with these data, after stimulation with serum THOC5 was recruited to 3′UTR of the *Id3* gene, but not the promoter region of *Id3* (Figure 6A) (24). THOC5 was not recruited to the THOC5-independent IEG, *Ier2* (Figure 6A). In control cells, CPSF100 was recruited to 3′UTR of the *Id3* and *Ier2* genes. In THOC5-depleted cells, CPSF100 was recruited to the *Ier2* gene to a similar extent as in control cells, while it was not recruited to the *Id3* gene (Figure 6B), suggesting that THOC5 plays a role in recruitment of CPSF100 to 3′UTR of its target gene. RNA polymerase II was equally recruited to both genes in the presence or absence of THOC5 (Figure 6C), suggesting that THOC5 participates in 3′end-processing, but not elongation of THOC5 target genes.

Katahira *et* *al*. (14) proposed that THOC5 affects alternative polyadenylation site choice by recruiting CFIm68 in HeLa cells. Here, CFIm68 is recruited to the promoter region of THOC5 target genes that are constitutively active. We next examined whether CFIm68 is also recruited to THOC5-dependent IEGs in the absence of THOC5. In agreement with previous data (14), CFIm68 was recruited to promoter regions of both genes. Strikingly, CFIm68 was also recruited to the *Id3* gene in the absence of THOC5 (Figure 6D), suggesting that the regulation of 3′end-processing of IEG may be different than in constitutively active genes.

## DISCUSSION

Eukaryotic protein coding genes are divided into two groups. One group is regulated by signals, the so called IEGs or inducible genes, and the other one is a group of genes which are constitutively active (such as housekeeping genes). It has been shown that transcriptional regulation of inducible genes may be different than that of housekeeping genes (25,26). RNA polymerase II is already present and poised for transcription at many inducible genes. Upon cell stimuli paused RNA polymerase II is released into productive transcription elongation (27). Moreover, it has been proposed that full-length LPS-induced nascent RNAs that contain introns appear to accumulate on chromatin, presumably to complete processing, prior to release of functional mRNAs and are then exported to the cytoplasm (19). However 3′end-cleavage and export of IEGs have not been well studied. In this case the THO complex was shown to play a role in 3′end-processing and nuclear export of IEG RNAs. We have previously identified THOC5-dependent mRNAs in the fibroblast system (11). Surprisingly, only 143 genes were downregulated by depletion of THOC5. Along the same line, depletion of THOC5 in HeLa cells does not affect bulk poly (A)+ RNA export (28) and it has been recently shown that the knockdown of THOC5 in HeLa cells leads to downregulation of 275 genes (14). Despite downregulation of <1% of genes upon THOC5 depletion, the knockdown of THOC5 in mice showed strong phenotypic traits. THOC5 depletion in adult mice causes suppression of hematopoiesis (29) and intestinal epithelial differentiation (15). On the other hand, we have previously shown that the depletion of THOC5 in differentiated organs such as liver did not cause any pathological alteration. In addition, in this organ constitutively expressing mRNAs such as albumin and transferrin mRNAs are THOC5-independent (15), suggesting that THOC5 plays a more important role in genes that are expressed transiently upon cell extrinsic and intrinsic signals which regulate cellular differentiation, proliferation and lineage specificity. We show here that although six THOC5 independent IEGs out of eight genes are involved in cell proliferation, more than 80% of the THOC5 target IEGs are relevant to the proliferation of MEF cells. In addition, using the bone marrow macrophage system we previously showed that genes with regulation of differentiation are apparently a target of THOC5 action (12). The depletion of THOC5 influences 3′end-processing, and mRNA export of most IEGs. In this context, one of the cleavage- and polyadenylation-specific factors, CPSF100 was recruited to the 3′end of THOC5 target IEG only in the presence of THOC5, however the mechanism by which THOC5 recruited CPSF100 to its target genes still remains to be studied.

Recently, Katahira *et* *al*. (14) proposed that THOC5 affects alternative polyadenylation site choice by recruiting cleavage factor I 68 (CFIm68 or CPSF6) on constitutively active THOC5 target genes, however the known THOC5 target mRNA, Hsp70 (8,11,13,30) and some of our identified THOC5 target genes do not contain an alternative polyadenylation site. Moreover, 70–75% of human mRNAs contains a potential alternative polyadenylation site (31). In this context, we compared the location of PAS hexamers and UGUA elements in THOC5-dependent and -independent genes, however we did not find any substantial differences between these genes. PAS can be repressed by non-coding small RNA such as micro RNA (32) or U1 snRNP (33).

Notably, THOC5-independent rapid IEGs do not contain introns (*Ier2, C8orf4, c-Jun*) or they are co-transcriptionally spliced (*Fos, FosB, Egr1, Zfp36, Btg2*) (19). It is presently not clear, however, whether splicing or introns influence THOC5 dependency.

These data indicate that IEG response is not only controlled by the transcriptional regulator, but also by the THO complex via 3′end-processing machinery.

## ACCESSION NUMBERS

The complete microarray data along with processing protocols have been deposited in NCBI's Gene Expression Omnibus and are accessible through GEO series accession number GSE59561.

## SUPPLEMENTARY DATA

Supplementary Data are available at NAR Online.

SUPPLEMENTARY DATA
