# Supplementary material for: THOC5 controls 3′end-processing of immediate early genes via interaction with polyadenylation specific factor 100 (CPSF100)
Source: Nucleic Acids Res. 2014 Oct 1;42(19):12249–60. doi: 10.1093/nar/gku911 (PMC4231767; doi:10.1093/nar/gku911)
Supplement: SUPPLEMENTARY DATA [file supp_42_19_12249__index.html]

THOC5 controls 3′end-processing of immediate early genes via interaction with polyadenylation specific factor 100 (CPSF100) — THOC5 controls 3′end-processing of immediate early genes via interaction with polyadenylation specific factor 100 (CPSF100) — SUPPLEMENTARY DATA 

# THOC5 controls 3′end-processing of immediate early genes via interaction with polyadenylation specific factor 100 (CPSF100)

## SUPPLEMENTARY DATA

**Files in this Data Supplement:**

- SUPPLEMENTARY DATA
