# Supplementary material for: THOC5 controls 3′end-processing of immediate early genes via interaction with polyadenylation specific factor 100 (CPSF100)
Source: Nucleic Acids Res. 2014 Oct 1;42(19):12249–60. doi: 10.1093/nar/gku911 (PMC4231767; doi:10.1093/nar/gku911)
Supplement: SUPPLEMENTARY DATA [file supp_gku911_nar-02200-x-2014-File008.pdf]

Supplementary Data, Tran et al.

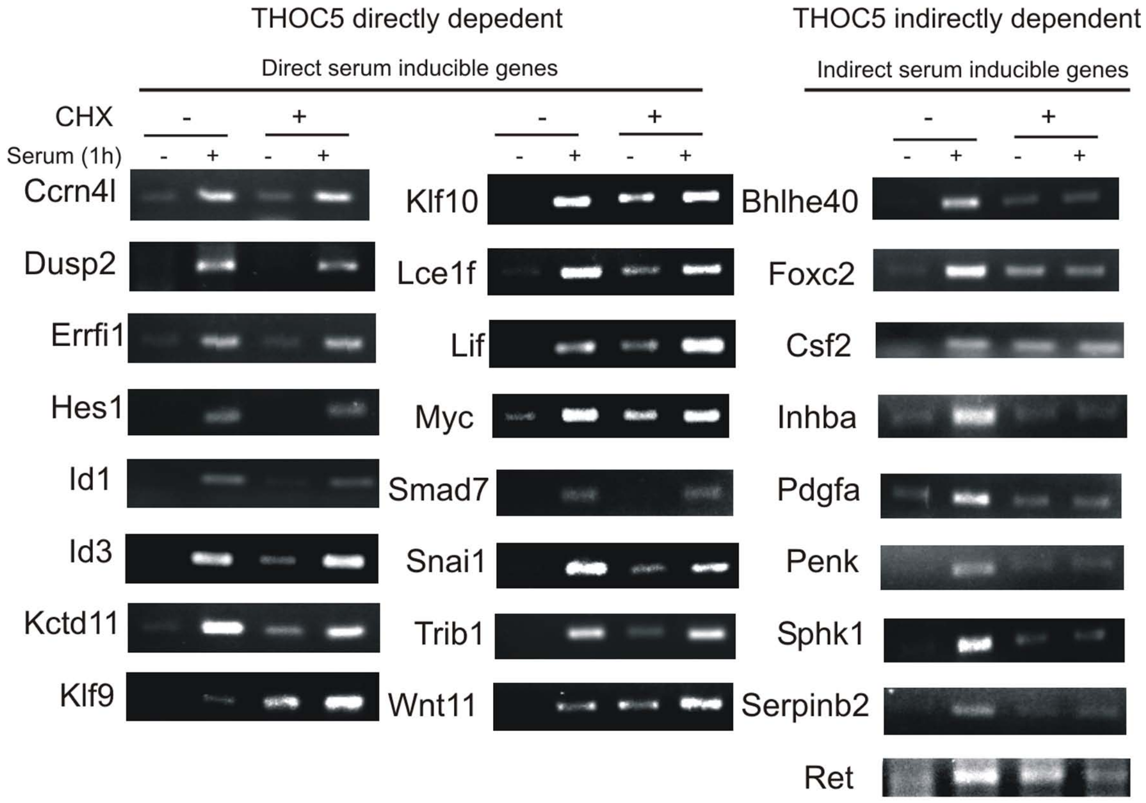

Figure S1: Identification of direct upregulated genes upon serum stimulation.

Cells were treated with or without cycloheximide and were then serum starved.

Before or after serum stimulation for 1h, RNAs were isolated and supplied for semi-quantitative RT-PCR.

PCR primers are shown in Table S2.

Table S1: List of 101 genes which are upregulated more than 4-fold:

Accession Number	Gene	Description
NM_001009819	A3galt2	alpha 1,3-galactosyltransferase 2 (isoglobotriaosylceramide synthase)
NM_178630	Agbl3	ATP/GTP binding protein-like 3
NM_009675	Aoc3	amine oxidase, copper containing 3
NM_031159	Apobec1	apolipoprotein B mRNA editing enzyme, catalytic polypeptide 1
ENSMUST00000089450	Apol8	apolipoprotein L 8
NM_018790	Arc	activity-regulated cytoskeleton-associated protein
NM_009704	Areg	amphiregulin
NM_029466	Arl5b	ADP-ribosylation factor-like 5B
NM_007498	Atf3	activating transcription factor 3
NM_011498	Bhlhe40	basic helix-loop-helix family, member e40
NM_007570	Btg2	BTG family, member 2
NM_001177577	C4orf26	chromosome 4 open reading frame 26
NM_026931	C8orf4	chromosome 8 open reading frame 4
NM_001081062	Ccno	cyclin O
NM_009834	Ccrn4l	CCR4 carbon catabolite repression 4-like (S. cerevisiae)
NM_007669	Cdkn1a	cyclin-dependent kinase inhibitor 1A (p21, Cip1)
NM_019952	Clcf1	cardiotrophin-like cytokine factor 1
NM_153582	Cmtm4	CKLF-like MARVEL transmembrane domain containing 4
NM_009969	Csf2	colony stimulating factor 2 (granulocyte-macrophage)
NM_009971	Csf3	colony stimulating factor 3 (granulocyte)
NM_153287	Csrnp1	cysteine-serine-rich nuclear protein 1
NM_010217	Ctgf	connective tissue growth factor
NM_001081335	Cul9	cullin 9
NM_008176	Cxcl1	chemokine (C-X-C motif) ligand 1
NM_010516	Cyr61	cysteine-rich, angiogenic inducer, 61
NM_010054	Dlx2	distal-less homeobox 2
NM_018808	Dnajb1	DnaJ (Hsp40) homolog, subfamily B, member 1
NM_010090	Dusp2	dual specificity phosphatase 2
NM_176933	Dusp4	dual specificity phosphatase 4
NM_001085390	Dusp5	dual specificity phosphatase 5
NM_026268	Dusp6	dual specificity phosphatase 6
NM_007913	Egr1	early growth response 1
NM_010118	Egr2	early growth response 2
NM_007950	Ereg	epiregulin
NM_133753	Errfi1	ERBB receptor feedback inhibitor 1
NM_010171	F3	coagulation factor III (thromboplastin, tissue factor)
NM_010194	Fes	feline sarcoma oncogene
NM_010234	Fos	FBJ murine osteosarcoma viral oncogene homolog
NM_008036	FosB	FBJ murine osteosarcoma viral oncogene homolog B
NM_013519	Foxc2	forkhead box C2 (MFH-1, mesenchyme forkhead 1)
NM_010276	Gem	GTP binding protein overexpressed in skeletal muscle
NM_008504	Gzmm	granzyme M (lymphocyte met-ase 1)
AK136956	Hapln3	hyaluronan and proteoglycan link protein 3
NM_008215	Has1	hyaluronan synthase 1
NM_010415	Hbegf	heparin-binding EGF-like growth factor
NM_008235	Hes1	hairy and enhancer of split 1, (Drosophila)
NM_010495	Id1	inhibitor of DNA binding 1, dominant negative helix-loop-helix protein
NM_010496	Id2	inhibitor of DNA binding 2, dominant negative helix-loop-helix protein
NM_008321	Id3	inhibitor of DNA binding 3, dominant negative helix-loop-helix protein
NM_031166	Id4	inhibitor of DNA binding 4, dominant negative helix-loop-helix protein
NM_010499	Ier2	immediate early response 2

NM_133662	Ier3	immediate early response 3
NM_010500	Ier5	immediate early response 5
NM_013562	Ifrd1	interferon-related developmental regulator 1
NM_008350	Il11	interleukin 11
NM_008380	Inhba	inhibin, beta A
NM_010577	Itga5	integrin, alpha 5 (fibronectin receptor, alpha polypeptide)
NM_008416	Junb	jun B proto-oncogene
NM_153143	Kctd11	potassium channel tetramerization domain containing 11
NM_001017426	Kdm6b	lysine (K)-specific demethylase 6B
NM_013692	Klf10	Kruppel-like factor 10
NM_010638	Klf9	Kruppel-like factor 9
NM_001033352	Klhl21	kelch-like family member 21
NM_026394	Lce1f	late cornified envelope 1F
NM_026335	Lce1h	late cornified envelope 1H
NM_029667	Lce1i	late cornified envelope 1I
NM_008501	Lif	leukemia inhibitory factor
NM_001033437	Lsmem1	leucine-rich single-pass membrane protein 1
NM_010755	Maff	v-maf avian musculoaponeurotic fibrosarcoma oncogene homolog F
NM_019471	Mmp10	matrix metallopeptidase 10 (stromelysin 2)
NM_010849	Myc	v-myc avian myelocytomatosis viral oncogene homolog
NM_010862	Myo15a	myosin XVA
NM_016791	Nfatc1	nuclear factor of activated T-cells, cytoplasmic, calcineurin-dependent 1
NM_153553	Npas4	neuronal PAS domain protein 4
NM_008726	Nppb	natriuretic peptide type B
NM_010444	Nr4a1	nuclear receptor subfamily 4, group A, member 1
NM_008808	Pdgfa	platelet-derived growth factor alpha polypeptide
NM_001002927	Penk	proenkephalin
NM_009344	Phlda1	pleckstrin homology-like domain, family A, member 1
NM_001045516	Proca1	protein interacting with cyclin A1
NM_011198	Ptgs2	prostaglandin-endoperoxide synthase 2 (prostaglandin G/H synthase and cyclooxygenase)
NM_019466	Rcan1	regulator of calcineurin 1
NM_001080780	Ret	ret proto-oncogene
NM_011267	Rgs16	regulator of G-protein signaling 16
NM_011111	Serpinb2	serpin peptidase inhibitor, clade B (ovalbumin), member 2
NM_008871	Serpine1	serpin peptidase inhibitor, clade E (nexin, plasminogen activator inhibitor type 1), member 1
NM_001161845	Sgk1	serum/glucocorticoid regulated kinase 1
NM_011386	Skil	SKI-like oncogene
NM_001042660	Smad7	SMAD family member 7
NM_011427	Snai1	snail family zinc finger 1
NM_011448	Sox9	SRY (sex determining region Y)-box 9
NM_025367	Sphk1	sphingosine kinase 1
NM_011898	Spry4	sprouty homolog 4 (Drosophila)
NM_025915	Tmem88	transmembrane protein 88
NM_001195710	Tmem95	transmembrane protein 95
NM_009397	Tnfaip3	tumor necrosis factor, alpha-induced protein 3
NM_144549	Trib1	tribbles homolog 1 (Drosophila)
NM_007887	Usp17l24	ubiquitin specific peptidase 17-like family member 24
NM_001025257	Vegfa	vascular endothelial growth factor A
NM_009519	Wnt11	wingless-type MMTV integration site family, member 11
NM_011756	Zfp36	ZFP36 zinc finger protein

Table S2. PCR primer pair sequences for cycloheximide treatment experiment.

Gene	Accession number	Forward primer	Reverse primer
Bhlhe40	NM_011498.4	GAAGCACGTGAAAGCATTGAC	AGTGACGAGCTGGGAAGATTT
Ccrn4l	NM_009834.2	CGCGTCATGCAGTGGAACATCC	CCCAGTCTACTGAGGAGTGGCTG
Csf2	NM_009969.4	CTTGGAAGCATGTAGAGGCCAT	CGTAGACCCTGCTCGAATATCT
Dusp2	NM_010090.2	TGTGGAAATCTTGCCCTACCT	CCCACTATTCTTCACCGAGTCTA
Errfi1	NM_133753.1	GCTGCTCAGGATATTCGAGTC	CCAACAGTTGTTAGGTGCTCC
Foxc2	NM_013519.2	CACCTCCTGGTATCTGAACCAC	GACGGCGTAGCTCGATAGG
Hes1	NM_008235.2	CCAGCCAGTGTCAACACGA	AATGCCGGGAGCTATCTTTCT
Id1	NM_010495.3	CTGAACTCGGAGTCTGAAGT	ACTTTTTTCCTCTTGCCTCCT
Id3	NM_008321.2	CGCATCTCCCGATCCAGACA	CTGGGTTAAGATCGAAGCTCATCC
Inhba	NM_008380.1	TGAGAGGATTTCTGTTGGCAAG	TGACATCGGGTCTCTTCTTCA
Kctd11	NM_153143.4	TCTCGGGGTACACCTGCAT	TCTAGCCGAAAATGTGGACCT
Klf10	NM_013692.3	CATCTGAAGGCCCACGTGAG	AGCTTCTTGGCTGATAGGTGG
Klf9	NM_010638.4	TGGAGAGTCCCGATGAGGATA	GAGGCGTGTTTCCCCTTCG
Lce1f	NM_026394.3	CACAGTCTTGCTCCTGAACAC	GCAGCATCCTCCAGAGCTAC
Lif	NM_008501.2	GCTGTATCGGATGGTCGCATA	CACAGACGGCAAAGCACATT
Myc	NM_001177352.1	GCTGGATTTCCTTTGGGCGT	CGCAACATAGGATGGAGAGCA
Pdgfa	NM_008808.3	CGATGAGGACCTGGGCTT	TTCTCGGGCACATGGTTAATG
Penk	NM_001002927.2	GGACTGCGCTAAATGCAGCTA	GAAGCCTCCGTACCGTTTCAT
Ret	NM_001080780.1	CAAGGCCTTACTGTCTGCACA	CGCTGAGGGTGAAACCATCC
Serpinb2	NM_011111.4	GCTCAACAATCAACACACCAC	GGTAGCAGGTTTGGGATTTCA
Smad7	NM_001042660.1	GCATTCCTCGGAAGTCAAGAG	CCAGGGGCCAGATAATTCGT
Snai1	NM_011427.2	CAAGGAGTACCTCAGCCTGG	GGTCAGCAAAAGCACGGTT
Sphk1	NM_001172472.1	TATGCTGGGTACGAGCAGGT	CCCACTGTGAAACGAATCTCC
Trib1	NM_144549.4	CCGAATCGCCGACTACCTG	CGCACATAGGAGTGCATGTCT
Wnt11	NM_009519.2	GGTGGTACACCGGCCTATG	TCACTGCCGTTGGAAGTCTTG

Bhlhe40: basic helix-loop-helix family, member e40 (217nt); **Ccrn4l**: CCR4 carbon catabolite repression 4-like (S. cerevisiae) (207nt); **Csf2**: colony stimulating factor 2 (granulocyte-macrophage) (157nt); **Dusp2**: dual specificity phosphatase 2 (232nt); **Errfi1**: ERBB receptor feedback inhibitor 1 (182nt); **Foxc2**: forkhead box C2 (192nt); **Hes1**: hairy and enhancer of split 1 (166nt); **Id1**: Inhibitor of DNA binding 1 (203nt); **Id3**: Inhibitor of DNA binding 3 (202nt); **Il1rl1**: interleukin 1 receptor-like 1 (202nt); **Inhba**: inhibin beta-A (183nt); **Kctd11**: potassium channel tetramerisation domain containing 11 (239nt); **Klf10**: Kruppel-like factor 10 (218nt); **Klf9**: Kruppel-like factor 9 (160nt); **Lce1f**: late cornified envelope 1F (242nt); **Lif**: leukemia inhibitory factor (156nt); **Myc**: myelocytomatosis oncogene (274nt); **Pdgfa**: platelet derived growth factor, alpha (232nt); **Penk**: preproenkephalin (274nt); **Ret**: ret proto-oncogene (199nt); **Serpinb2**: serine (or cysteine) peptidase inhibitor, clade B, member 2 (226nt); **Smad7**: SMAD family member 7 (225nt); **Snai1**: snail family zinc finger 1 (179nt); **Sphk1**: sphingosine kinase 1 (218nt); **Trib1**: tribbles homolog 1 (249nt); **Wnt11**: wingless-related MMTV integration site 11 (183nt).

Table S3: A list of the proteins identified via mass spectrometry from the gel walk of the TAP-TAG THOC5 immunoprecipitation in the presence and absence of serum:

Gene name	Number of peptides seen in sample without serum	Number of peptides seen in sample with serum	Gene name	Number of peptides seen in sample without serum	Number of peptides seen in sample with serum
AAAS	3	1	CDC42EP1	2	1
AAMP	0	1	CDIPT	1	0
AARS	14	5	CEBPZ	1	0
AARS2	8	6	CEP57L1;C6orf182	1	0
ABCD3	0	1	CEPT1	0	1
ABCE1	2	1	CFH;CFHR1	1	0
ABCF2	6	0	CHD1L	4	1
ABCF3	2	0	CLPX	0	1
ABI1	1	0	CLPX;DKFZp586J151	5	0
ACACB	46	27	CNOT1	2	0
ACAD11	2	1	COG5	1	0
ACADM	7	0	COQ6	1	0
ACOT9	2	0	CP	7	0
ACSL3	2	0	CPNE7;CPNE6;CPNE5;CPNE8;CPNE4;CPNE9;CPNE2;CPNE3	1	0
ADAR	6	3	CPSF100	0	1
ADSL	1	0	CPSF73	1	0
ADSS	1	0	CRSP3;MED23	1	0
AFG3L2	5	7	CRTAP	2	0
AGK	3	0	CSTA	0	1
AGPAT5	1	0	CTNNBL1	2	0
AGPS	1	0	CTNND1	4	1
AHNAK	4	1	CTPS	12	3
AHSA1	3	0	CTSD	4	0
AKAP1	1	0	CUL4A	2	0
AKAP8	1	1	CUL5	0	1
AKAP8L	3	4	CYFIP1;CYFIP2	4	2
ALDH1B1	6	2	DDI1	0	1
AMPD2	8	10	DDOST	7	0
ANXA1	0	4	DDX10	3	0
APMAP	1	0	DDX12P;DDX11L8;DDX12;DDX11	0	2
APOB	1	0	DDX19B;DDX19A	2	0
ARG1	1	0	DDX20	3	2
ARHGEF2	2	1	DDX23	2	1
ARMC6	2	0	DDX47	1	0
ASNS	2	3	DDX54	4	7
ATAD3B	19	9	DDX56	2	0
ATP13A1	1	0	DHCR7	1	0
ATP1A1	12	6	DHX30	8	12
ATP6V1H	2	1	DIS3	1	2
ATR	0	1	DKC1	0	1
ATXN10	4	0	DLAT	3	3
BAZ1B	0	2	DLST	3	2
BCCIP	3	0	DMAP1	1	1
BCKDHA	3	0	DMD	1	1
BTAF1	3	1	DNAJB12	1	0
BYSL	2	0	DNAJC11	1	0
C20orf4	1	0	DNAJC7	3	2
C22orf28	1	2	DNM1L	2	0
C4B-1;C4A;C4B	1	0	DNM2	10	0
C6orf211	2	0	DNM3;DNM2;DNM1	0	2
CALML3	1	0	DSC1	3	1
CALML5	5	2	DTNA	0	1
CALU	1	0	DUS3L	0	2
CAND2	0	2	DUSP9	1	0
CAPN2	2	0	DYNC1LI1	0	2
CASP14	2	2	DYNC1LI2	0	1
CBS	4	0	DYNC2H1	1	0
CCDC47	3	1	EARS2	2	2

ECM29;KIAA0368	10	8	IDH1	3	0
EHD4	4	0	IDH3B	1	0
EHD4;EHD3	0	2	IGHA1;IGHA2	1	0
EIF2B3	1	0	IGHG1;IGHG3;IGHG4;IGHG2	2	0
EIF2B4	1	0	IKBKAP	4	5
EIF2B5	1	2	IMMT	17	16
EIF4G2	0	2	IPO11	1	0
ERAL1	1	0	IPO4	6	6
ERLIN1;ERLIN2	2	0	IPO7	6	7
ERVK6;ERVK-6;ERVK-5	0	1	IPO9	3	5
ESYT2	1	0	IRAK1;NEK2	2	0
EXOC1	2	1	IRS2	0	1
EXOC4	3	0	ISYNA1	0	1
FABP5	2	2	ITIH2	2	0
FAM105B	1	0	KIAA0090	2	0
FAM120A	1	0	KIF14	1	1
FANCI	4	5	KIF3A	1	0
FAR1	2	0	KLHL17	1	0
FARSA	5	5	KNG1	1	0
FDFT1	1	0	KPNA1	1	1
FDXR	2	0	KPNA3	0	1
FHDC1	1	0	KPNA6	1	0
FHOD1	2	1	LAS1L	1	0
FIGNL1;VPS4B;VPS4A	1	0	LEMD2	2	0
FKBP8	2	0	LGALS7	4	2
FN1	3	0	LMAN1	3	2
FOXK1	2	1	LMF2	1	0
FTSJ3	4	8	LUZP1	0	1
FXR1	4	1	LYZ	1	0
GAK	0	1	MAK16	1	0
GALK1	2	0	MAP2K7	1	0
GAPVD1	3	0	MAPK1	1	0
GARS	2	0	MAPK3	2	0
GBP1	0	1	MARK2	0	1
GCDH	2	0	MAT2A	2	0
GEMIN4	4	2	MDC1	4	6
GFPT1;GFPT2	2	0	MDN1	10	4
GGCT	0	1	MMS19	3	1
GIGYF2	0	3	MOCS3	1	0
GLS	5	0	MPP6	1	0
GMPPA	2	0	MRPL37	2	0
GMPS	3	0	MRPL38	2	0
GNAS;GNAL;GNAI2;GNAT3;GNAI3;GNAO1;GNAT2;GNAI1;GNAT1	1	0	MRPS22	3	0
GNL2	1	3	MRPS27	2	0
GOPC	3	8	MSH2	17	7
GPATCH4	2	1	MSH6	11	11
GPN1	1	0	MTOR	3	0
GRWD1	3	1	MUC19	0	1
GSPT2;GSPT1	2	0	MYLK2	2	2
GSR	2	0	MYO1D	2	0
GTF3C1	2	0	MYO6	1	3
GTF3C2	2	0	NAA15	1	0
GTPBP10	1	0	NAMPT	2	0
GTPBP4	4	0	NCAPD2	5	5
HADHB	7	0	NCAPG	0	1
HEATR1	3	3	NCAPH	1	0
HEATR2	4	3	NCBP1	2	1
HELLS	5	4	NCLN	4	2
HELZ	3	2	NDUFA10	2	0
HK1	6	3	NDUFS1	7	1
HK2	6	2	NDUFV1	3	0
HM13	1	0	NEFL	18	13
HNRNPUL2	0	2	NEURL4	0	1
HPSE	1	0	NKRF	1	0
HPX	2	0	NOB1	2	0
HSDL2	3	0	NOC2L	4	2
IARS2	5	0	NOC3L	2	0

NOC4L	2	0	SDHA	2	0
NOL6	0	2	SEC31A	1	0
NOP14	3	0	SEC61A1;SEC61A2	2	0
NPEPPS;NPEPPSL1	2	2	SERPINA3	1	0
NRBP2	0	1	SHROOM3	0	2
NSF	7	2	SLC16A1	1	2
NSUN2	8	7	SLC22A13	1	0
NSUN4	2	0	SLC25A12	7	3
NUP107	6	0	SLC25A13	11	6
NUP133	1	0	SLC2A1	0	1
NUP155	8	9	SLC3A2	2	0
NUP160	1	4	SLFN11	2	0
NUP188	2	7	SLTM	0	1
NUP205	14	8	SMARCA5	3	8
NUP88	1	0	SMARCB1	1	0
NUP93	12	10	SNX2	2	0
OGFR	1	0	SNX4	0	1
OGT	1	0	SNX6	2	0
OPA1	5	1	SPATA5	0	2
OR3A3;OR3A2	1	0	SPTLC1	3	1
OSBPL8	1	0	SRPK2	0	1
OXSR1	3	0	SRPR	1	0
PANK4	0	1	STAT1	1	2
PAX2	0	1	STAU1	0	2
PDCD11	5	3	STK4	0	1
PDCD4	2	0	STOML2	4	0
PDCD6IP	5	2	STT3A	3	0
PDHA1	2	0	SUCLA2	4	0
PDHX	0	2	SUCLG2	2	0
PDS5A	2	3	SUGP2	2	2
PELP1	1	0	SURF4	1	1
PES1	0	1	SUZ12	0	1
PFAS	4	5	SYCP1	1	0
PLCXD2	0	1	SYNE1	0	1
PLG	1	0	TARS2	6	4
PNN	0	3	TBC1D10B	0	1
POLD1	7	6	TBCD	3	3
POLD3	1	0	TBRG4	3	0
POLR1C	2	0	TELO2	6	0
POLR2A;SLC35G6	1	0	TET3	0	1
POLR2B	4	3	TFIP11	2	1
POLR3A	0	2	TGM3	0	2
POP1	0	7	THOC1	7	17
POR	1	0	THOC2	12	20
PPA2	0	1	THOC3	2	0
PPAN-P2RY11;PPAN	1	2	THOC5	36	35
PPM1G	2	0	TIMM44	2	0
PPP6R1	1	1	TMEM48	1	0
RBM14	11	3	TNKS	0	1
RBM15	1	0	TNPO1	5	0
RBM28	5	4	TOMM40	1	0
RCC1	3	0	TOP2B	2	5
RFC2	3	0	TRIP13	4	0
RGPD3	7	6	TROVE2	3	0
RNF40	6	5	TSFM	1	0
RNH1	1	0	TTC27	3	1
RPAP1	2	0	TTC37	2	2
RRM1	2	0	TTLL12	9	3
RRP12	5	4	TUBGCP2	0	2
RRP15	1	0	TUBGCP3	4	0
RRP1B	2	5	TXNDC5	1	0
RTCD1	1	0	UBE2O	1	0
S100A7	2	0	UBE3C	3	1
S100A8	4	4	UBTF	3	1
S100A9	4	3	UMPS	2	3
SAMHD1	3	3	UNC45A	1	0
SCAMP4	0	1	UPF1	1	0
SDF4	2	0	UQCRC1	6	0

UQCRC2	9	0	VTN	3	0
USO1	5	1	XAB2	1	1
USP10	2	1	XPO5	5	7
USP5	1	0	XPO7	3	1
UTP14A	1	0	XPOT	8	2
UTP20	0	2	XRN1	0	5
VPS35	1	0	YTHDC2	1	4
VPS4A	0	2	ZRANB1	1	1
VPS52	2	4	ZSCAN29	1	0
VPS53	2	0	ZW10	1	0

This table was compiled with the use of a list of known non-specific interactor proteins (www.crapome.org). A threshold value for the use of the crapome.org data was set by reference to known interactors of THOC5, namely THOC1, 2, and 3.
